# Multiple Human Tracking Using Binary Infrared Sensors

**DOI:** 10.3390/s150613459

**Published:** 2015-06-08

**Authors:** Toshiaki Miyazaki, Yuki Kasama

**Affiliations:** The University of Aizu, Aizuwakamatsu, Fukushima 965-8580, Japan; E-Mail: m5151122@gmail.com

**Keywords:** infrared sensor, multiple human tracking, privacy

## Abstract

To create a context-aware environment, human locations and movement paths must be considered. In this paper, we propose an algorithm that tracks human movement paths using only binary sensed data obtained by infrared (IR) sensors attached to the ceiling of a room. Our algorithm can estimate multiple human movement paths without *a priori* knowledge of the number of humans in the room. By repeating predictions and estimations of human positions and links from the previous human positions to the estimated ones at each time period, human movement paths can be estimated. Simulation-based evaluation results show that our algorithm can dynamically trace human movement paths.

## 1. Introduction

Human tracking technologies have attracted considerable attention over the years. For example, with real-time human tracking technology, air conditioners and lights can be controlled smartly by considering the requirements of each human. In addition, elderly people or children can be monitored for safety reasons [1,2]. Consequently, many human tracking systems and algorithms have been proposed [3,4,5,6,7,8,9,10,11,12,13,14,15,16,17,18,19,20]. One popular method involves camera-based systems [3,4,5,6,7,8,9,10]. However, they are often not acceptable for monitoring elderly people or other similar applications whose objective is to observe humans without invading their privacy. This is because people may be uncomfortable with monitoring using video cameras, even if the original image data are modified and not used directly to perform human tracking. In addition, the size of video data is large compared to other sensor data; therefore, camera-based systems are not suitable for long-term observations. In other popular methods, IC tags or radio frequency identification devices (RFIDs) [11,12] are used. A person wears a RFID, which is then detected by nearby RFID receivers located on the floor or ceiling of the monitored area. Thus, using data stored in the RFID receivers, the person can be tracked. Although the RFID-based systems can be considered to enhance privacy, they force people to carry the RFID devices.

For these reasons, some human tracking methods use infrared (IR) sensors [13,14,15,16,17,18,19,20]. The IR sensor produces a “1” if it detects a human, and a “0” otherwise [21]. Thus, using the IR sensors, the movement paths of subjects can be estimated without sacrificing their privacy. In addition, IR sensors are low-cost and their installation is relatively simple. However, IR sensors have a deficiency—they react regardless of whether they detect one or multiple persons. Therefore, almost all human tracking methods using IR sensors assume that the number of humans in the room is known beforehand. However, from the perspective of practical application development, the number of humans in a room is often unknown.

We previously proposed an algorithm that can simultaneously estimate both the number of humans and their movement paths [22,23], however, the method still had some drawbacks. Thus, the algorithm is invoked after all sensed data are collected, making it difficult to estimate the human movement in real time. In addition, the estimated human position must be the position of a fired IR sensor or the midpoint of the overlapped detection areas of multiple fired IR sensors; thus, the method lacks flexibility. To address these issues we now propose a novel method that can overcome the aforementioned problems [24]. The method dynamically estimates human positions using the weighted centers of grouped fired IR sensors, instead of simply using the position of a fired IR sensor or the midpoint of the overlapped detection areas of multiple fired IR sensors in the previous methods. Thus, by using the new method, we can estimate multiple human movement paths in a timely manner, and the estimated human positions are not restricted by the locations of the IR sensors, thus improving the human tracking accuracy.

The remainder of this paper is organized as follows: in Section 2, a model of the assumed IR sensor system is described. Next, the details of the proposed algorithm are explained in Section 3. Then, evaluation results are shown in Section 4. Finally, our conclusions and future studies are described in Section 5.

## 2. Model of IR Sensor System

In this paper, we consider a system constructed using IR sensors and a PC. The IR sensors are randomly installed on the ceiling of a room, but their locations are known. In addition, each IR sensor has a non-directional and circular detection range, the radius of which is *r.* Here, *r* is typically several meters, if we use commercially available IR sensors [21]. We assume *r* = 2.0 m in this paper. The sensor outputs binary data, *i.e.*, it produces a “1” if it detects one or more humans and a “0” otherwise. Moreover, all the IR sensors are connected to the PC using a wired or wireless communication network. Thus, data obtained from the IR sensors are collected in the PC. The data sampling rate is 6 Hz, and the sensed data are saved on the PC with a time stamp. In addition, the positional coordinates of each sensor is known. Furthermore, the detection ranges of the IR sensors should cover the entire area to detect human movements. Note that some IR sensor arrays have been proposed recently [25], and they can detect human movements, but their detection range is typically several meters. Thus, even if using the IR sensor array, it is difficult to cover a large area. Furthermore, the method proposed in this paper can naturally handle the IR sensor array by treating it as a set of densely deployed IR sensors, if the sensor array produces a set of {0, 1}-data depending on human detection.

## 3. Proposed Algorithm

We can trace a human movement path by connecting the positions of the fired sensors step by step, if the position coordinates of each sensor is known. However, this method presents the problem that if some of the IR sensors are close together, they often detect the same event and output “1” data at the same time, which could make it difficult to track the human movements using the simple method mentioned above. This is especially true when there is more than one person in the room. To solve this problem, we propose a heuristic algorithm that can estimate multiple human movement paths using only the binary sensed data.

### 3.1. Notations

First, to explain the procedures of our algorithm, we define the variables and parameters as follows:

∙ |*|: The number of elements in list *.
∙ **CC**(t): A set of the weighted center coordinates of the clusters at time t. **CC**(t) = {cc_i_(t)|i = 1, …, C_max_(t)}, where cc_i_(t) = (x, y) and they are referred to as cc_i_(t).x and cc_i_(t).y, respectively.
∙ C_max_(t): The number of clusters at time t.
∙ d_s_(t): A binary sensed datum obtained by IR sensor s at time t. d_s_(t) ϵ {0, 1}.
∙ md_AVE_(td.ID): An average of movement distance of target human “td.ID.” The details are described later.
∙ **mv**_t_(td.ID): A coordinate set of the most recently estimated WS2 number of coordinates in **Path** of “td.ID” at time t. **mv**_t_(td.ID) = {mc_i_(td.ID)|i = 1, 2, … WS2}, where mc_i_(td.ID) = (x, y). They are referred to as mc_i_(td.ID).x and mc_i_(td.ID).y, respectively. mc_1_(td.ID) is more recent than mc_2_(td.ID).
∙ ***MV***(td.ID): A vector from coordinate mc_WS2_(td.ID) to coordinate mc_1_(td.ID), *i.e.*, mc_WS2_(td.ID) → mc_1_(td.ID). This vector is referred to as the movement vector of target human “td.ID” at time t.
∙ **ORD**(t): A binary dataset obtained by applying the logical-OR operation to **RDL** at time t. **ORD**(t) = {o_s_(t)|s = 1, …, S}, where o_s_(t) = ${\text{U}}_{\text{i}=t}^{\text{t}-\text{WS}1+1}{\text{d}}_{\text{s}}(\text{i}),\text{s}=1,...,\text{S}$.
∙ *r*: The radius of the detection range of each IR sensor.
∙ **RD**(t): A set of sensed raw data at time t. **RD**(t) = {d_s_(t)|s = 1, …, S}. |**RD**(t)| = S.
∙ **RDL**: A list containing sets of the sensed raw data. At time t, **RDL** = {**RD**(t-WS1 + 1), **RD**(t-WS1 + 2), …, **RD**(t-1), **RD**(t)}. |**RDL**| = WS1.
∙ S: The total number of IR sensors. S > 0.
∙ td: A data structure representing a target human. It contains ID, TTL, PC, and **Path**. They are referred to as td.ID, td.TTL, td.PC, and td.**Path**, respectively.
∙ td.ID: A human ID. This is an integer value. Td.ID > 0.
∙ td.PC: A predicted coordinate of target human “td.ID” at time t, where td.PC = (x, y). They are referred to as td.PC.x and td.PC.y, respectively.
∙ td.**Path**: A list of the estimated route coordinates of target human “td.ID.” By connecting all elements in this list from the first to the last, the estimated movement path of human “td.ID” is obtained.
∙ td.TTL: A lifetime of target human “td.ID”. This is an integer value. The initial value of td.TTL is “1,” and its maximum value is TTL_MAX_.
∙ **TDL**: A list of target humans who currently exist in the room.
∙ TTL_MAX_: A constant integer value. The maximum value of TTL (time to live). TTL_MAX_ > 0.
∙ **WL**(t): A weight set of the sensors at time t. **WL**(t) ={w_s_(t)|s = 1, …, S}.
∙ w_s_(t): A weight of sensor s at time t. w_s_(t) = ${\text{∑}}_{\text{i}=\text{t}}^{\text{t}-\text{WS}1+1}{\text{d}}_{\text{s}}(\text{i})|\text{s}=1,...,\text{S}$. This is used to calculate the weighted center coordinate of a cluster, to which sensor s belongs. The coordinate calculation method will be defined later.
∙ WS1: Window size for the number of sensed raw datasets to which the logical-OR operation is applied. This is a given integer value. WS1 > 0.
∙ WS2: Window size or the number of elements (coordinates) that construct movement vector **mv**_t_(td.ID). This is a given integer value. WS2 > 1.

### 3.2. Main Procedure

**Figure 1:**
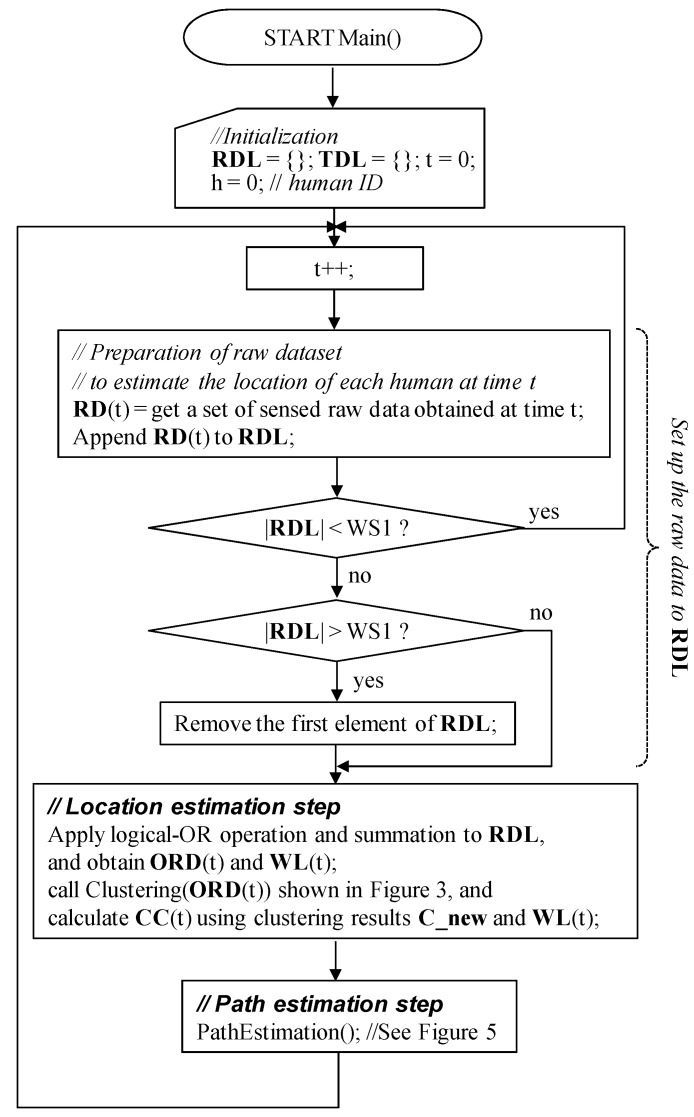
Main procedure of the proposed algorithm.

The main procedure of the proposed algorithm is shown in Figure 1. It mainly consists of two processing steps: the *location estimation* step and *path estimation* step. The former is described as a part of the main procedure, whereas the latter step will be shown later. At each time t, the two steps are invoked sequentially, and a link from the current to the next human position is estimated. First, as the *location estimation* step, the candidates for human positions are listed using a clustering technique. Then, in the *path estimation* step, the links are determined by selecting reasonable positions among the candidates obtained in the *location estimation* step. At time t, the position of each human is estimated using the candidate sets, *i.e.*, the sets of the weighted centers of clusters, obtained from t-WS2 + 1 to t. Here, WS2 is relatively small. Thus, our algorithm can trace the human movement paths almost in real time, without knowing the number of humans in the room. Our previous algorithm could not do this [22,23]. In addition, the framework of the proposed algorithm is the same as that of the algorithm introduced in [24], except that both the *location estimation* and *path estimation* steps are modified to improve human tracking accuracy. In the following subsections, the detailed procedure of each step will be explained.

### 3.3. Location Estimation Step

**Figure 2:**
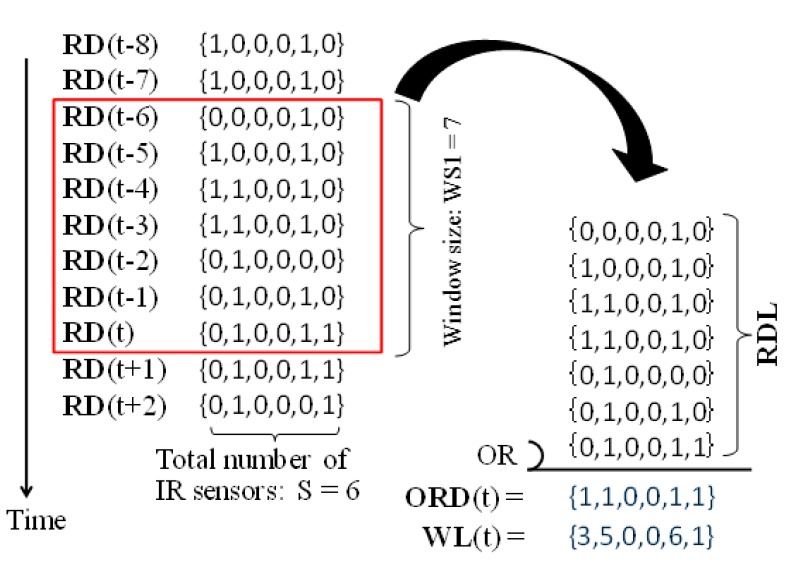
Example of sensed raw data processing.

The main purpose of this step is to provide a candidate list containing the next locations of the humans in the room. To achieve this, logical-OR operations and summations are applied to list **RDL** that contains all sensed raw data in time period [t, t-WS1 + 1], and **ORD**(t) and **WL**(t) are obtained. Figure 2 shows an example. In this example, S = 6, and WS1 = 7. **RDL** = {(0,0,0,0,1,0), (1,0,0,0,1,0), …, (0,1,0,0,1,1)}.

Thus, **ORD**(t) becomes {1,1,0,0,1,1} after applying a logical-OR operation to **RDL**, and **WL**(t) becomes {3,5,0,0,6,1} by summing up the corresponding sensed raw data in **RDL**. Here, in the case of S = 1, we cannot estimate human movements actually. However, we can detect if humans exist in the detection area of the IR sensor. It is often called “proximity” position detection. To simplify the discussion, we treat S = 1 case as a human-trackable case in this paper.

**Figure 3:**
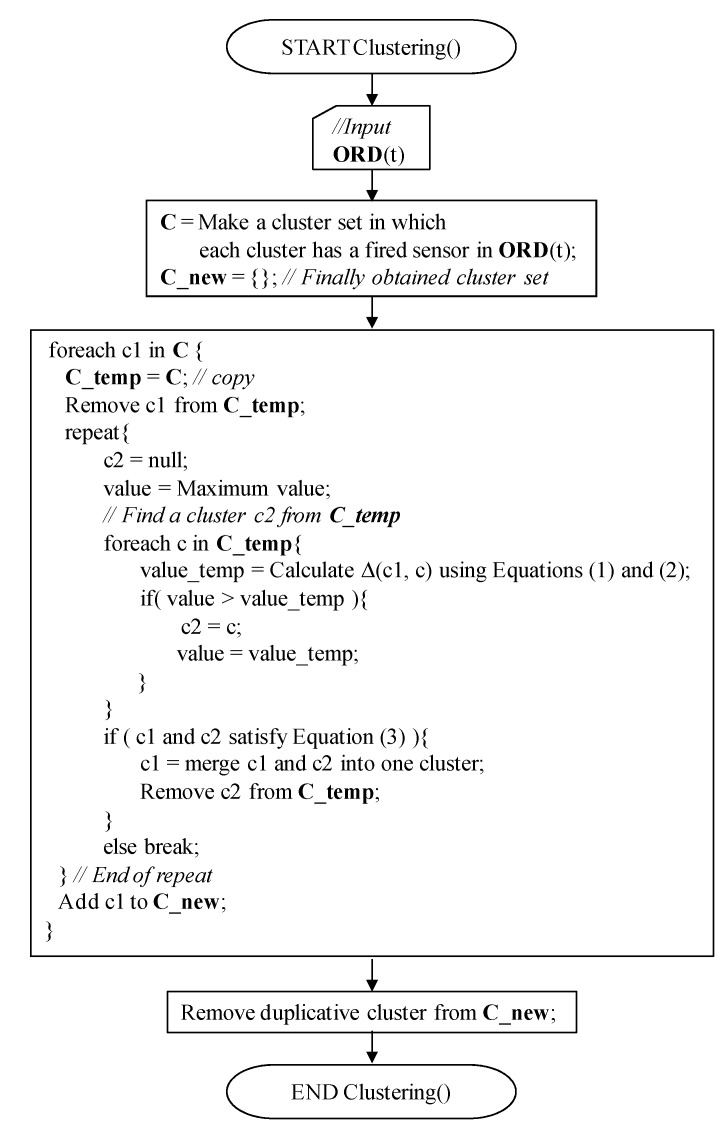
Clustering algorithm.

Next, using **ORD**(t) and **WL**(t), the fired sensors during time period [t, t-WS1 + 1] are grouped using a clustering method. Finally, the weighted centers of the clusters are calculated, and they are used in the *path estimation* step as the candidates for the next human positions. The clustering algorithm is shown in Figure 3. The algorithm is based on the Ward method [26]; however, the termination condition of our algorithm is different from that of the original method. While the original Ward method tries to merge the nearest pair of clusters until all initial clusters are merged into one cluster, our algorithm stops the cluster merging if Equation (3), *i.e.*, the termination condition, is no longer satisfied. The geometrical distance between the centers of any two clusters in the finally obtained cluster set will be longer than the radius of the detection range of IR sensor *r*.

Here, we assume that a person exists in each obtained cluster, and his/her position is the weighted center of the cluster. The weighted center coordinate cc_i_(t) = (x_i_(t), y_i_(t)) of cluster c_i_(t) at time t is calculated using Equation (4).

**Figure 4:**
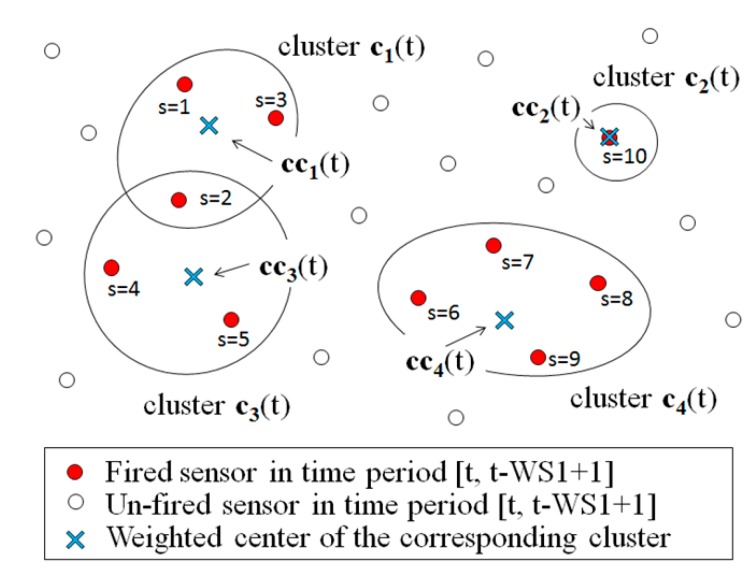
Example of clustering.

Note that each element can belong to more than one cluster in our new clustering method, contrary to that used in our previous algorithm [24]. Thus, we can manage the case that multiple humans exist in the detection range of the same IR sensor, which cannot be managed by our previous algorithm introduced in [24]:

(1) $$\underset{\forall c_{i},\forall c_{j}\in C;i\neq j}{\min}\Delta (c_{i},c_{j})=E(c_{i}\cup c_{j})-E(c_{j})-E(c_{j})$$

(2) $$E(c)=\sum_{\forall k\in c}\left\{{\left(x_{k}-\bar{X}\right)}^{2}+{\left(y_{k}-\bar{Y}\right)}^{2}\right\}$$

$$\bar{X}=\frac{1}{\left|c\right|}\sum_{\forall k\in c}x_{k}\text{, }\bar{Y}=\frac{1}{\left|c\right|}\sum_{\forall k\in c}y_{k}$$

where $\text{(}x_{k},y_{k}\text{)}$ is the coordinate of element k, and |c| is the number of elements in cluster c:

(3) $$r^{2}<\frac{1}{\left|c_{i}\cup c_{j}\right|}E(c_{i}\cup c_{j})$$

(4) $$x_{i}(t)=\frac{\sum_{\forall s\in c_{i}(t)}w_{s}(t)\cdot x_{s}(t)}{\sum_{\forall s\in c_{i}(t)}w_{s}(t)},\;y_{i}(t)=\frac{\sum_{\forall s\in c_{i}(t)}w_{s}(t)\cdot y_{s}(t)}{\sum_{\forall s\in c_{i}(t)}w_{s}(t)}$$

where “s” is a sensor ID. The weighted centers of all clusters are set to **CC**(t). They become candidates for human positions at time t. Figure 4 shows an example. In the example, cluster c_1_(t) consists of sensors s = 1, s = 2, and s = 3. If the position coordinates of the sensors are (10, 10), (9, 5), and (14, 8), and their weights are w_1_(t) = 4, w_2_(t) = 1, and w_3_(t) = 2, the center coordinate cc_1_(t) will be (11.0, 8.7), *i.e.*, x_1_(t) = (4 × 10 + 1 × 9 + 2 × 14)/(4 + 1 + 2) and y_1_(t) = (4 × 10 + 1 × 5 + 2 × 8)/(4 + 1 + 2). Furthermore, sensor s = 2 belongs to another cluster c_3_(t). Thus, the coordinate of s = 2 is also used to calculate the weighted center cc_3_(t) of cluster c_3_(t). If a cluster contains only one sensor, like cluster c_2_(t) in Figure 4, the center position coordinate is simply the position of the sensor. After all weighted center coordinates of the clusters are calculated and set to **CC**(t), the *path estimation* step will be initiated.

### 3.4. Path Estimation Step

**Figure 5:**
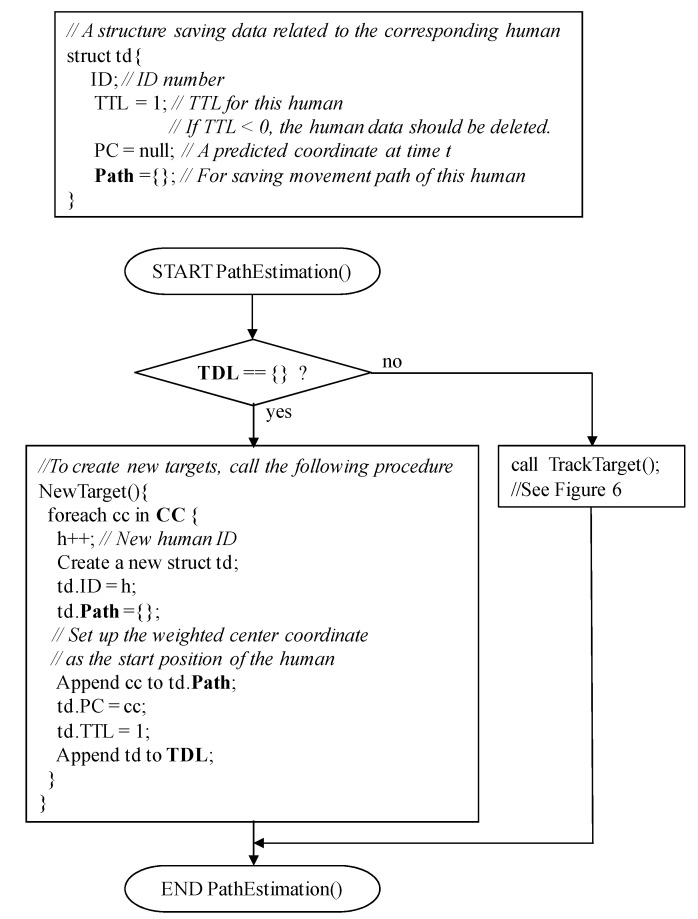
Procedure of the *path estimation* step.

**Figure 6:**
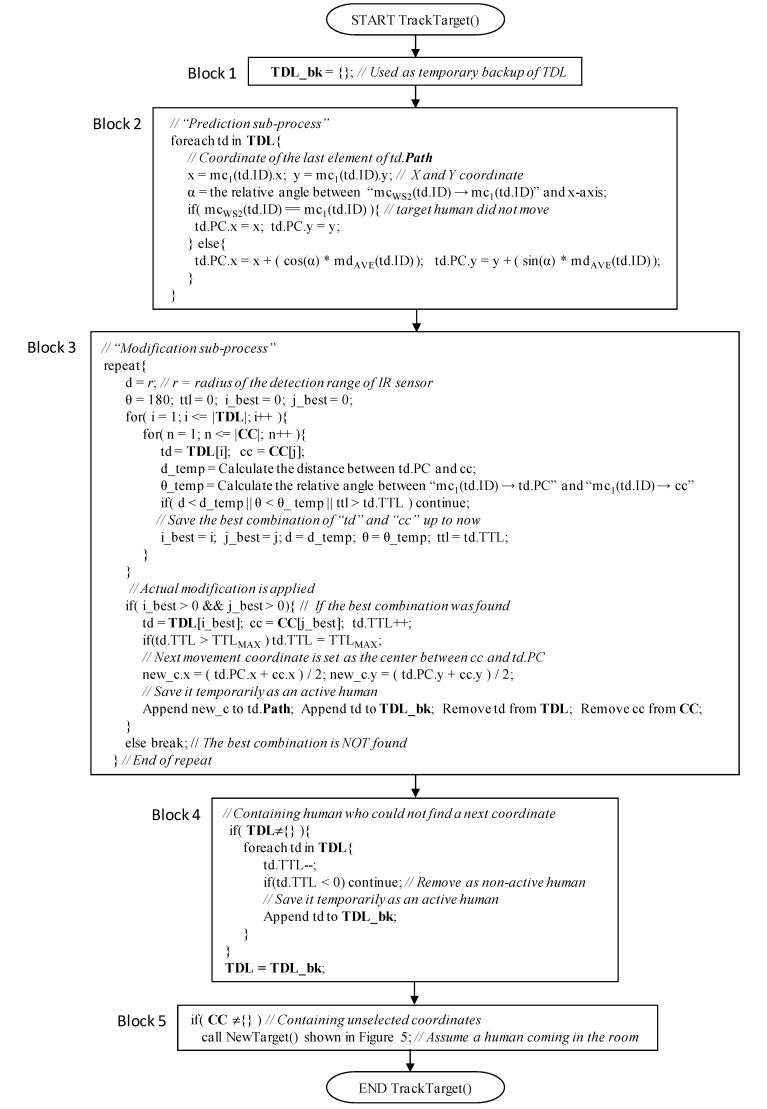
Procedure to estimate the next position of each human.

**Figure 7:**
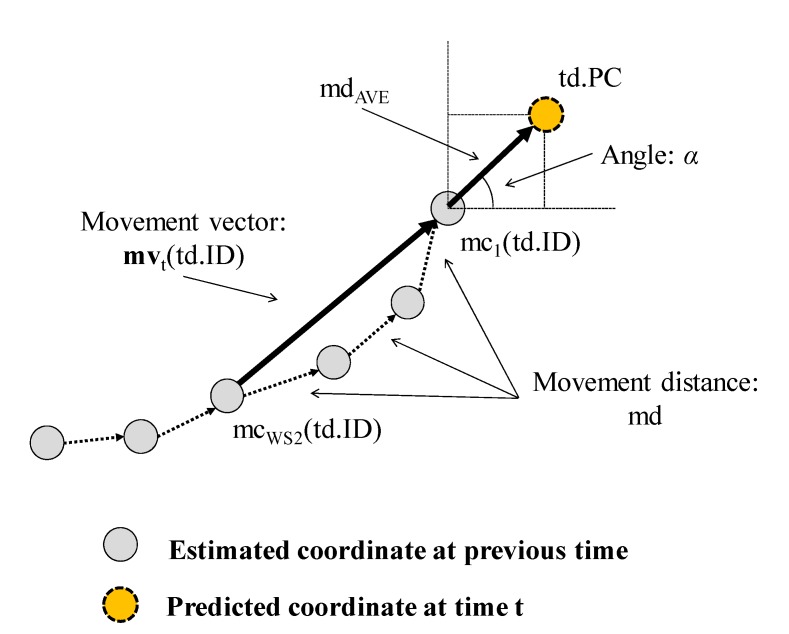
Example of the calculation of predicted next coordinate.

The procedure of this step is described in detail in Figure 5 and Figure 6. In the main procedure, PathEstimation(), if **TDL** is empty, NewTarget() is called to create new humans. Their starting points are simply the weighted center coordinate of **CC**, respectively. Here, **TDL** represents humans currently existing in the room. If some humans exist, *i.e.*, **TDL** is not empty, TrackTarget() in Figure 6 is invoked to obtain the next position coordinate of each human. TrackTarget(), which has been changed from our previous one introduced in [24], contains two sub-processes: *prediction* and *modification*. In the *prediction* sub-process, a predicted coordinate, td.PC, is provided for each human as the next position coordinate at time t (see “Block 2” in Figure 6). First, a movement angle *α* of each human at time t is calculated using a movement vector ***MV***(td.ID). Here, the movement vector represents the expected direction of the corresponding human, and is calculated using the most recently estimated WS2 number of the position coordinates of the human, *i.e.*, **mv**_t_(td.ID). Here, equal values for mc_WS2_(td.ID) and mc_1_(td.ID) indicate that the target human did not move. Therefore, the predicted coordinate td.PC is simply treated as mc_1_(td.ID). Otherwise, the prediction is calculated. Figure 7 shows an example. First, an average of movement distance md_AVE_(td.ID) is calculated using **mv**_t_(td.ID), defined as:

(5) $$md_{AVE}(td.ID)=\frac{1}{WS2-1}\sum_{i=2}^{WS2}\sqrt{{\left(x\prime_{i-1}-x\prime_{i}\right)}^{2}+{\left(y\prime_{i-1}-y\prime_{i}\right)}^{2}}$$

where *x’_i_* = mc_i_(td.ID).x and *y’_i_* = mc_i_(td.ID).y. Using the movement angle *α* and md_AVE_(td.ID), the predicted coordinate *td.PC* is calculated (see also procedure for TrackTarget() shown in Figure 6).

Next, as the *modification* sub-process, all pairs of the weighted coordinates in **CC** and target humans in **TDL** are examined, and the best pair is selected in consideration of distance *d*, angle *θ*, and time to live (TTL) of “td.” Here, “*d*” is the distance between td.PC and cc. “*θ*” is the relative angle between “mc_1_(td.ID) → td.PC” and “mc_1_(td.ID) → cc.” A pair that minimizes “*d*” and “*θ*” and maximizes TTL at the same time is finally selected as the best pair.

Actual *modification* is, then, applied for the best pair found in the *prediction* sub-process, and the next movement position coordinate is obtained as the center of td.PC and cc of the best pair (see also “Block 3” in Figure 6). The obtained next position is appended to the td.**Path** of the corresponding target human. The elements representing the selected pair of the weighted center coordinate cc and target td are removed from **CC** and **TDL**. The aforementioned best-pair selection process is repeated until no more selected pairs exist. In addition, if the next position of a human can be successfully found, the TTL value of the human is incremented; otherwise, it is decremented. Here, the initial value of TTL is “1,” and its maximum value is TTL_MAX_. The TTL value never exceeds TTL_MAX_. If the TTL of a human becomes less than 0, the human is removed from the **TDL** as he/she has left the room. In addition, if an unselected coordinate is contained in **CC**, NewTarget() is invoked and a human is appended to **TDL** as he/she enters the room. Due to this mechanism, even if the number of humans is not estimated correctly using the clustering algorithm in the *location estimation* step shown in Figure 1 and Figure 3, and some humans have accidentally been generated by NewTarget() at this point, they will be removed within a short period.

**Figure 8:**
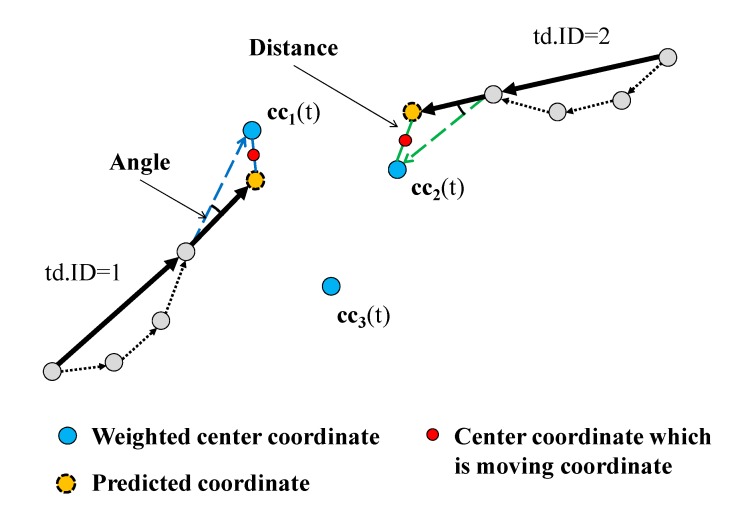
Example of TrackTarget().

Figure 8 shows an example of the *Path Estimation* step. Here, **TDL** = {td1, td2} and **CC** = {cc_1_(t), cc_2_(t), cc_3_(t)}. First, a pair of td1.pc and cc_1_(t) is selected. The distance between them is smaller than that between others, and the angle is less than 180° and smaller than that between others. Thus, the center coordinate of td1.PC and cc_1_(t) is calculated, and it is appended to td1.**Path**. In addition, td1.TTL is incremented. In the next step, because **TDL** = {td2} and **CC** = {cc_2_(t), cc_3_(t)}, a pair of td2.PC and cc_2_(t) is selected, and the process described above is applied to the pair.

Our path estimation algorithm shown in Figure 6 can be formulated using Equations (6) and (7). Assume that ${\text{p}}_{\text{t}}$ is the position of a target human at time t, ${\text{p}}_{\text{t}}=\left[\begin{array}{cc} {\text{x}}_{\text{t}} & {\text{y}}_{\text{t}} \end{array}\right]$. Then, a prediction at time t is given by:

(6) $${\hat{p}}_{t}=p_{t-1}+Bu_{t}$$

where ${\hat{\text{p}}}_{\text{t}}$ is a predicted coordinate at time t; in this paper, it is the same as td.PC: ${\hat{\text{p}}}_{\text{t}}\;=\left[\begin{array}{cc} {\text{x}}_{\text{t}} & {\text{y}}_{\text{t}} \end{array}\right]$. In addition, B is the average of the movement distance md_AVE_(td.ID) of the target human, and ${\text{u}}_{\text{t}}$ is the x-y component form of the predicted movement distance at time t. ${\text{u}}_{\text{t}}=\left[\begin{array}{cc} \sin\alpha & \cos\alpha \end{array}\right]$. Here, *α* is the angle of the movement vector ***MV***(td.ID) at time t. Furthermore, the modified position at time t is given by:

(7) $$p_{t}=\frac{1}{2}({\hat{p}}_{t}+z_{t})$$

where ${\text{z}}_{\text{t}}$ is a measurement of the target human at time t and is the same as the center position coordinate cc(t), which is obtained by the best-pair selection. ${\text{z}}_{\text{t}}=\left[\begin{array}{cc} {\text{x}}_{\text{t}} & {\text{y}}_{\text{t}} \end{array}\right]$.

Equation (6) is similar in form to the Kalman filter, which is used in related work [19,20]. However, as compared to the original Kalman filter, in our proposed algorithm, ${\hat{\text{p}}}_{\text{t}}$ and ${\text{z}}_{\text{t}}$ are calculated as mentioned previously, not using the method for obtaining the Kalman gain. Since the system environment is drastically changed by the numbers of IR sensors and humans in a room and the detection range and distribution density of IR sensors, we cannot apply the calculation method to obtain the Kalman gain.

## 4. Evaluation

### 4.1. Evaluation Environment

To evaluate our algorithm, we used generated simulation data. The details are as follows. First, the density of IR sensors “D” is defined by:

(8) $$\text{D}=\frac{\text{S}\cdot \text{π}\cdot r^{2}}{\text{A}}$$

where “S” is the number of IR sensors; “*r*” is the radius of the IR sensor detection range, *r* = 2.0 m; and “A” is the entire area of the room monitored, A = 100 m^2^ (10 m × 10 m). We assume that the entire area A should be covered with the minimum number of IR sensors. Thus, if we fix the density D as 2.0, 3.0, 4.0, or 5.0, the number of IR sensors S that should be deployed is calculated using Equation (8). The corresponding value of S is actually 16, 24, 32, or 40 as shown in Table 1. Here, “S” sensors should be randomly deployed in the area.

**Table 1:** Number of deployed IR sensors S used for experiments. It is related to the density of the sensors D.

D	S
2.0	16
3.0	24
4.0	32
5.0	40

*r* = 2.0 m

The number of humans “H” is changed from one to four, and the human movement data are created for each case. The human movement scenario is as follows. After a random waiting time from 0 to 30 s, each human should enter an area through a door and walk around randomly. The walking scenarios are as follows. The human decides a destination position and walks toward it using the shortest path. Then, the human either stops at the destination position, or walks to a new random destination. Here, the human does not return quickly. After 60 s, the human should exit through the door. The walking speed of each human is randomly changed from 1.25 m/s to 1.75 m/s. For each “H,” 50 different variations of data were generated. In total, 800 (4 different H × 4 different S × 50 different variations) different input data were generated and used for the evaluation of the proposed algorithm. Here, the parameters of our algorithm are WS1 = 4, WS2 = 24, and TTL_MAX_ = 18. According to our preliminary estimations, these combinations of values produce good path estimations.

### 4.2. Evaluation Results and Remarks

Our method estimates the number of humans and their movement paths simultaneously. Thus, it is difficult to evaluate the method using tracking errors like in other human tracking methods, which assume that the number of humans is fixed and known [20,27]. To evaluate our method, we introduced four metrics: success estimation rate, averaged error, averaged tracking rate, and success rate of the number of humans. The details are as follows: Table 2, Table 3, Table 4 and Table 5 show the evaluation results. For comparison, we also evaluated the previously proposed algorithm [24]. In the tables, the upper values represent the results of the proposed algorithm and the lower ones represent the results of the previous one.

#### (1)   Success estimation rate

Table 2 represents the percentage of 50 input patterns provided for each different case that were estimated correctly by our algorithm. For H = 1, both algorithms estimated all human movement paths correctly for all different densities. For H = 2, the success estimation rate of the proposed and previous algorithms were 60% and 57% on average, respectively. In addition, for H = 3 and H = 4, both the estimation rates were decreased to less than 34%. If the number of humans increase, their movement paths are often overlapped, which makes it difficult to estimate individual human movement. Thus, the success estimation rate decreases with the increase in the number of humans in a room, regardless of the path estimation methods being compared.

**Table 2:** Success estimation rate (Unit: %).

Density (D)	Number of Humans (H)			
	H = 1	H = 2	H = 3	H = 4
D = 2.0	100	56	34	6
	100	66	28	6
D = 3.0	100	64	22	4
	100	60	32	4
D = 4.0	100	64	24	10
	100	46	20	10
D = 5.0	100	56	28	14
	100	56	22	6
Average	100.0	60.0	27.0	8.5
	100.0	57.0	25.5	6.5

Upper value: The result obtained by the proposed algorithm; Lower value The result obtained by the previous algorithm [24].

#### (2)   Averaged error

Table 3 shows the averaged error of the estimated human movement paths compared to the true human movement paths among the successfully estimated data. In our algorithm, a position coordinate of the IR sensor was basically used. Therefore, an error in the detection range of the IR sensor is contained during the initial stage of the path estimation. Even so, for H = 1 and H = 2, the proposed method can trace the human movement path accurately for less than 0.60 m and 1.42 m on average, respectively. However, although our previous algorithm can trace one human for less than 0.68 m, the averaged error is more than 2.08 m when H = 2. In other cases, it could not trace the human movement paths correctly.

#### (3)   Averaged tracking rate

In Table 4, the averaged tracking rate for each target human is described. The tracking rate is defined as the ratio of the length of the correctly estimated route to the total length of the actual human movement path. If the rate is high, it indicates that the human path is estimated well for a long time. For H = 1, the human was tracked with an accuracy rate of more than 94% in both algorithms. In other cases, if the density was high, the tracking rate was also high. This implies that many sensors are needed to estimate multiple humans.

**Table 3:** Averaged error of the estimated path (Unit: m).

Density (D)	Number of Humans (H)			
	H = 1	H = 2	H = 3	H = 4
D = 2.0	0.60 (0.32)	1.22 (0.89)	2.05 (1.50)	3.09 (2.09)
	0.68 (0.36)	2.08 (1.69)	3.11 (2.23)	3.42 (2.28)
D = 3.0	0.59 (0.29)	1.27 (0.88)	1.97 (1.43)	1.52 (1.04)
	0.66 (0.34)	2.24 (1.73)	3.12 (2.27)	3.90 (2.32)
D = 4.0	0.59 (0.31)	1.42 (0.94)	1.44 (1.02)	2.04 (1.43)
	0.66 (0.35)	2.43 (1.84)	3.21 (2.22)	3.18 (2.26)
D = 5.0	0.54 (0.27)	1.38 (1.11)	2.01 (1.42)	2.62 (1.63)
	0.61 (0.30)	3.20 (2.35)	3.03 (2.26)	2.69 (2.39)
Average	0.58 (0.30)	1.32 (0.96)	1.87 (1.34)	2.32 (1.55)
	0.65 (0.34)	2.49 (1.90)	3.12 (2.25)	3.30 (2.31)

Upper value: The result obtained by the proposed algorithm; Lower value The result obtained by the previous algorithm [24]; ( ): standard deviation.

**Table 4:** Averaged tracking rate (Unit: %).

Density (D)	Number of Humans (H)			
	H = 1	H = 2	H = 3	H = 4
D = 2.0	97.98 (7.33)	57.22 (14.04)	35.40 (5.60)	33.56 (2.73)
	94.63 (13.31)	61.34 (17.19)	43.30 (8.87)	42.90 (2.38)
D = 3.0	99.78 (0.09)	66.94 (16.34)	55.12 (11.15)	43.47 (5.13)
	99.55 (0.10)	69.67 (12.97)	58.22 (8.06)	54.60 (1.34)
D = 4.0	99.78 (0.06)	75.39 (13.19)	58.46 (9.13)	48.46 (5.11)
	99.55 (0.07)	71.77 (15.41)	54.64 (4.07)	55.06 (7.89)
D = 5.0	99.78 (0.07)	83.20 (14.34)	64.51 (14.57)	51.57 (6.82)
	99.55 (0.07)	73.69 (13.02)	59.29 (4.40)	58.39 (2.70)
Average	99.33 (1.89)	70.69 (14.48)	53.37 (10.11)	44.27 (4.95)
	98.32 (3.39)	69.12 (14.65)	53.86 (6.35)	52.74 (3.58)

Upper value: The result obtained by the proposed algorithm; Lower value The result obtained by the previous algorithm [24]; ( ): standard deviation.

#### (4)   Success rate of the number of humans

Table 5 shows what percentage of the number of humans correctly estimated in the room at each time. It is directly related to our clustering algorithm. Using the proposed algorithm, when H = 1 and H = 2, the success rates of H were more than 97% and 76%, respectively. However, for H = 3 and H = 4, these rates were from 45% to 60%. If the number of humans increases, the probability that some of them are walking close together increases. Therefore, the performance of the clustering algorithm decreases. Compared to our previous algorithm, the newly proposed clustering algorithm does not perform well.

**Table 5:** Success detection rate of number of humans (Unit: %).

Density (D)	Number of Humans (H)			
	H = 1	H = 2	H = 3	H = 4
D = 2.0	97.76 (0.95)	83.15 (5.58)	60.32 (5.58)	52.79 (1.81)
	98.87 (0.64)	85.79 (6.36)	59.77 (6.62)	44.63 (4.07)
D = 3.0	97.87 (1.01)	76.76 (9.58)	58.97 (8.43)	55.69 (6.29)
	99.03 (0.59)	84.17 (9.64)	69.65 (9.13)	50.08 (0.08)
D = 4.0	98.86 (0.54)	81.62 (8.53)	55.01 (9.76)	51.20 (1.83)
	99.54 (0.31)	88.17 (8.39)	61.91 (11.76)	54.41 (3.68)
D = 5.0	99.10 (0.38)	78.22 (6.65)	57.73 (7.74)	45.62 (3.49)
	99.68 (0.17)	88.83 (6.35)	71.43 (7.61)	53.90 (9.94)
Average	98.40 (0.72)	79.94 (7.59)	58.01 (7.88)	51.13 (3.35)
	99.28 (0.43)	86.74 (7.68)	65.69 (8.78)	50.76 (4.44)

Upper value: The result obtained by the proposed algorithm; Lower value The result obtained by the previous algorithm [24]; ( ): standard deviation.

According to these results, if the number of humans in the room increased, the tracking accuracy of our method decreased. This is because our clustering algorithm does not work well if some humans walk closely together. In addition, if relatively many humans exist, e.g., when H = 3 and H = 4 in our experiments, almost all the IR sensors are fired simultaneously. In such cases, no algorithm can estimate the number of humans correctly. However, as shown for H = 2, our algorithm can estimate the human movement paths well with a 1.42 m averaged error. In addition, our human-location-estimation algorithm based on a clustering method performs with a success rate of greater than 76% even for the complicated case when two persons enter and exit the room at different times. Compared to our previous algorithm, the clustering method itself in the proposed algorithm does not perform well, because the new clustering method allows a position candidate to belong to more than one cluster. However, owing to this fact, the averaged accuracy of the final human tracking is improved. As shown in Table 3, the averaged errors of the estimated human movement paths are improved two-fold for H = 2.

**Figure 9:**
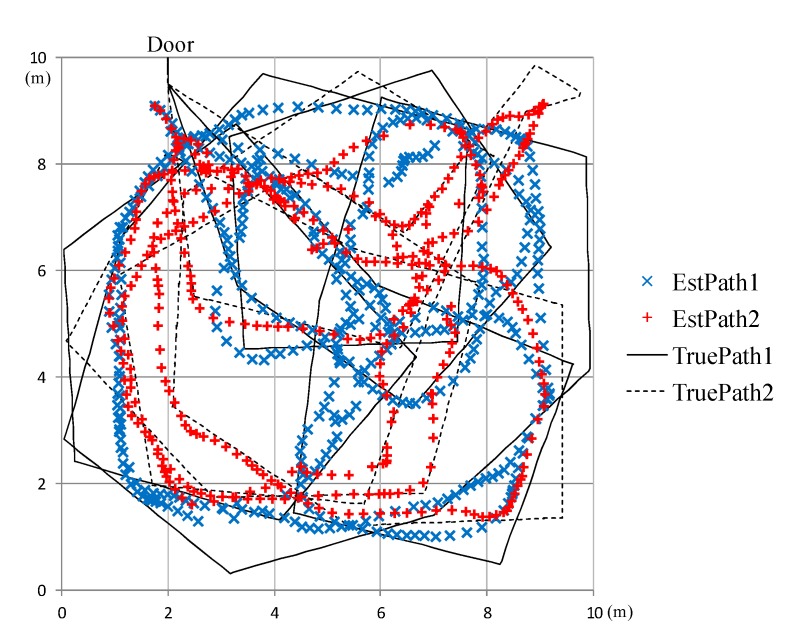
Example of the path estimation results.

Figure 9 illustrates an example of the estimated human movement paths for H = 2. The solid line is the true path of the first human, and the dotted line is the true path of the second human. They are indicated by “TruePath1” and “TruePath2” in Figure 9. Two different kinds of markers represent the estimated locations of two persons, respectively. They are “EstPath1” and “EstPath2” in the figure. The start and goal locations of both persons are the same, which is indicated as “Door” in the figure. As shown in Figure 9, our proposed algorithm can trace human movement well. This is the best result among 50 different input data generated with the same condition, D = 5.0. That is, 40 sensors were deployed in the field. The numerical results for the case shown in Figure 9 are as follows: the averaged error of the estimated paths, corresponding to Table 3, is 0.56 m. The averaged tracking error, corresponding to Table 4, is 99.89%. Success detection rate of number of humans, corresponding to Table 5, is 86.85%.

## 5. Conclusions

We have proposed an algorithm that can track the human movement paths using only the binary sensing data obtained from infrared sensors attached to the ceiling. The human positions are estimated at each timepoint based on a clustering method. Thus, the proposed algorithm can track multiple humans even if the number of humans in the room is changed dynamically, which was difficult to realize using methods proposed in related studies. According to simulation-based evaluations, our algorithm can trace real human movement paths with a 1.32 m error on average if two humans are in the room. In future studies, we will evaluate our algorithm using real sensed data obtained from a real infrared sensor system.
